# Anesthetic Considerations in Athletes: A Review

**DOI:** 10.7759/cureus.81040

**Published:** 2025-03-23

**Authors:** Mohd Batchi, Hashaam Ghafoor, Anwar Ul Huda, Ali O. Mohamed Bel Khair, Osman Ahmed, Ekambaram Karunakaran, Siddalingappa Suresh Orekondi, Shameen Salavudheen, Mohamed Sheriff Poolakundan, Jagadish Adiga, Tafazzul Husain, Aisha Abdalraheem Hamad Elawad, Ahmed Hussein Mohamed Almaqadma

**Affiliations:** 1 Department of Anesthesia and Perioperative Medicine, Hamad Medical Corporation, Doha, QAT; 2 Department of Anesthesia, Qatar University, Doha, QAT

**Keywords:** anesthesia consideration, athlete, complications, performance enhancing drugs, sport medicine

## Abstract

Athletes present a unique challenge for anesthesiologists because of their specific physiology and the demands of their sport. Anesthesia for athletes requires careful consideration of factors such as muscle mass, hydration status, and the potential for drug interactions with performance-enhancing substances. Additionally, the use of specific anesthetic agents, such as inhalational and intravenous anesthetics, requires adjustments to the dosing regimen to ensure adequate anesthesia. Sports anesthesia is a subspecialty that encompasses not only expert knowledge regarding regional and general anesthesia and pain management but also the pathophysiology unique to athletes and psychological concerns associated with professional sports and complete knowledge about World Anti-Doping Agency guidelines as well as substance abuse. Moreover, the pressure of being scrutinized by a broad circle of concerned persons, including family members, team members, coaches, club owners, media, and fans, makes this an area of excellence and expertise. This review highlights how an athlete’s physiological changes can alter anesthetic drug effects. Considering the importance of vigilant preoperative assessment, selecting the proper anesthesia plan, and perioperative strategies for better recovery and performance outcomes.

## Introduction and background

The practice of anesthesia has evolved into a diverse number of specialties that each address the specific requirements of a surgical discipline. These specialties include cardiac surgery, obstetrics, neurosurgery, pediatrics, burn management, maxillofacial surgery, neonatal care, trauma management, ambulatory surgery, and the emerging subspecialty of sports anesthesia [1], which refers to a comprehensive approach to providing perioperative care for athletes, from initial presentation at the pre-anesthesia clinic through surgical intervention and the postoperative period, which extends beyond discharge from the healthcare facility. This anesthesia specialty area requires expertise in regional and general anesthetic techniques as well as meticulous pain management, a thorough understanding of the athlete’s unique pathophysiology, the psychological stressors and anxieties inherent in competitive sports, and comprehensive knowledge of the World Anti-Doping Agency (WADA) guidelines and potential for substance misuse [1].

While many elite athletes possess a phenotype of high physical fitness and good health derived from intensive training and disciplined lifestyles, the possibility of underlying medical disease or conditions becomes evident during the preadmission assessment [1]. One of the widely accepted standards for care, owing to their universal requirement, independent of activity level considerations, is this review of already present conditions, such as asthma, diabetes mellitus, and endocrine disorders, that require meticulous assessment and optimization before the administration of any anesthetic agent [1].

This critical pre-anesthetic assessment often entails an exhaustive assessment of active medications that need adjusting to optimize their effectiveness and safety throughout the perioperative period. Further blood investigations, advanced imaging, or functional pulmonary tests may be necessary to obtain a detailed picture of the physiological status of the athlete and possible anesthetic risks [1]. If any other undiagnosed pathologies are suspected or identified, referring to other specialties must be warranted for investigation and optimization. This is an educational review focusing on the pharmacokinetics and pharmacodynamics of elite (competing) and non-elite (recreational) athletes, giving insight into anesthesia considerations for this specific group of people. In this study, we review the role of sports anesthesia and the various aspects related to good practice.

## Review

Pharmacokinetics of anesthetic drugs

Athletes typically exhibit a low body fat percentage and a high muscle mass relative to their total body weight (TBW). However, although widely used, the body mass index (BMI) does not provide a quantitative assessment of body composition or fat distribution. Consequently, individuals exhibiting elevated body weight secondary to increased muscle mass may be classified as obese according to the BMI criteria; nevertheless, these individuals might not exhibit the pathophysiological sequelae associated with excess adipose tissue [2]. Therefore, the clinical implications of body weight in this population depend on individual patient- and drug-specific factors. Specifically, a drug’s lipid or water solubility is a critical determinant, and its pharmacokinetic effects depend on the compositions of the relevant body compartments [2].

Effects of weight on body composition

**Figure 1:**
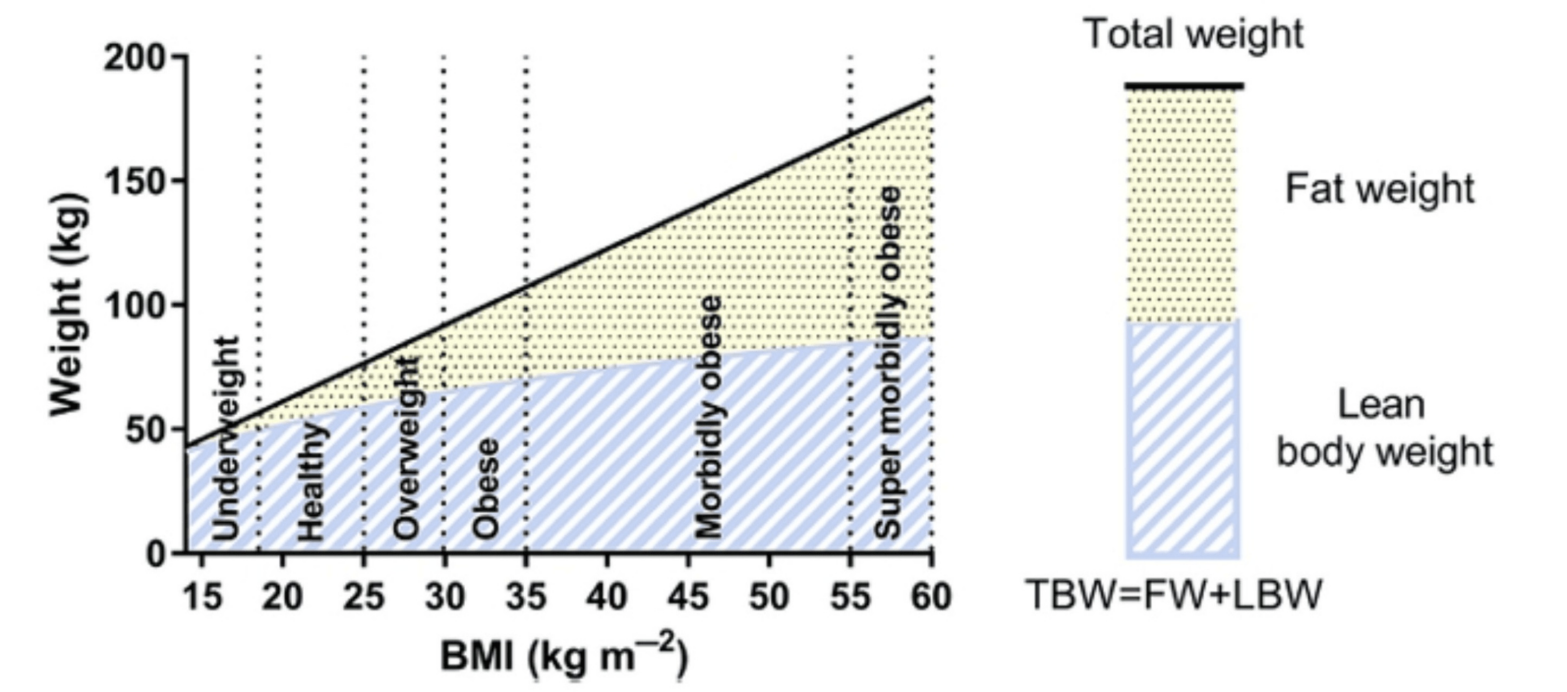
Changes in the relative proportion of FW, LBW, and TBW with increasing BMI for a male 1.75 m in height FW: fat weight; LBW: lean body weight; TBW: total body weight Credit: Permission to reproduce this image has been obtained from reference [2].

In individuals with normal body weight, TBW comprises 20% fat weight (FW) and 80% lean body weight (LBW). In malnourished and underweight persons, the lines for LBW and TBW converge because FW decreases the amount of TBW (Figure 1) [2].

LBW and protein-calorie malnutrition cause catabolism, lean mass, and fat loss, which affect protein binding, drug delivery, and clearance. The fatty tissue loss during starvation is highest at the start; however, with intensifying starvation, lean tissues and muscle are gradually lost [2]. As TBW increases in an obese individual, LBW increases relatively slowly because most of the additional weight is contributed by adipose tissue; thus, a relative increase is observed in the LBW/TBW ratio (Figure 1) and fat-free mass index. With increasing body weight, total body water is enhanced as well; however, in severe obesity, the total proportion of body water is decreased because the maximum additional weight is fat [3].

In the scientific literature, there is insufficient data available supporting the effects of acute malnutrition and low body weight on body composition. However, as LBW and TBW converge, the comparative fatty mass decreases (Figure 1) [2]. FW contains poorly vascularized adipose tissue wherein lipid-soluble medications could be sequestered, mainly with repeated doses or protracted management; such medications have an elevated volume of distribution (Vd) at a stable state. It is particularly relevant during critical care in which repeated administration or protracted infusion over many days of midazolam, propofol, morphine, and several other medications can cause a considerably delayed offset. After the discontinuation of the protracted infusion, as the plasma/effect-site concentrations decrease, drugs are delivered in the plasma as well as to their effect sites from the fatty tissues, extending the effect and delaying the terminal elimination phase. These effects are more obvious among obese individuals with a high FW [2].

The LBW is mainly muscle and vessel-rich organs/tissues, wherein drug delivery and eradication are comparatively fast. Lean tissues are extremely active, whereas the LBW proportion is associated with the basal metabolic amount, drug metabolism, and clearance. The blood supply to fat is 5% of the cardiac output compared with 22% to lean tissues [4,5]. The weight implications regarding clinical practice depend upon individual patient- and drug-related determinants. The water or lipid solubility of a medication is an important factor, but the outcomes depend on the body compartment composition. Most water-soluble medicines have low Vds, in contrast to the more lipid-soluble medicines that have elevated Vds [2].

Applied pharmacology

Numerous measures based on height, weight, and sex are used to calculate the drug dose [6-9]. Despite these measures, TBW remains insufficient for explaining some modifications in the compartment size. Therefore, derived markers estimating the compartment size (i.e., ideal body weight) or including a correction regarding drug delivery are preferable, particularly where the concentration of a drug is critical. Metabolism and distribution of adipose tissue differ between females and males; therefore, several measures exhibit sex differences [2].

Another technique is to modify the dosages per the LBW, which correlates well with the clearance of drugs. Among non-obese athletes, TBW is estimated from the LBW and is so frequently used with no adjustment. However, with increasing weight, the FW relative proportion is notably enhanced; hence, the LBW has been found to be much less than the TBW (Figure 1). Among obese individuals, it is generally preferable to use LBW, either presuming distribution only to lean tissues or extra fatty mass to describe the drug’s lipid solubility. The anticipated normal weight adjusts for extra adipose tissue and includes weight, height, and sex [2].

Pharmacokinetic changes

Gastrointestinal tract function is mostly preserved among obese individuals without considerable differences in digestion/first-pass metabolism. However, among athletes with low body weight, gastrointestinal absorption is reduced [2]. Among people with obesity, the pharmacokinetic alterations in the drug distribution are induced by alterations in size, plasma protein binding, and tissue-compartment composition. Enhanced volumes of the peripheral compartment can accumulate drugs in the poorly perfused tissues of obese individuals; for example, fentanyl late offset after long infusion is caused by saturation of inadequately perfused peripheral compartments [10].

In general, metabolism depends on the site (renal, hepatic, and several other sites), drug distribution, and extraction ratio. Although in obese individuals, renal and hepatic blood flow and cardiac output are all increased, drug distribution for metabolism within these body organs is higher. Hence, the clearance, as well as metabolism for flow-limited medications with an elevated extraction ratio (e.g., ketamine, propofol, and morphine), is enhanced. However, metabolic pathways could be repressed through the effects of fatty liver or concomitant chronic renal disease or enhanced due to the effects of several drugs, alcohol, and smoking. The effects of obesity are inconsistent on hepatic metabolism [2].

Drug clearance has been shown to be directly associated with LBW which is enhanced in obese individuals and is decreased in underweight people due to catabolism. The increase in the cardiac output associated with obesity enhances the glomerular filtration rate. During undernourishment, the glomerular filtration rate is comparatively well preserved until the late stages [2].

Specific examples of anesthesia

The effect of an IV anesthetic agent is assessed by its effect-site absorption, which successively depends upon the initial dosage, Vd, and lipid solvability. Given that both Vd and cardiac output are enhanced during obesity, an elevated bolus dosage is probably needed to attain a specified effect. The enhanced clearance and redistribution are associated with LBW; hence, LBW is suggested for use in bolus dosing of individuals with obesity [11].

The Marsh and Schnider models are typically used to determine the procedure for target-controlled infusion of propofol. Marsh’s model is only based on the individual’s overall weight and could lead to overdosing until an ideal body weight is used. The Schnider model uses age, sex, weight, and height to obtain compartment sizes and to assess the ke0 value (it is the proportional variation of the concentration gradient between plasma and effect site in relation to the unit of time*)* regarding patients; this causes lesser levels of overshoot as well as cardiovascular volatility. This model presumes a fixed size of the central compartment, whereas Marsh’s model adjusts this to the patient’s weight, leading to considerable differences between models regarding dosages to persuade anesthesia [2].

Another example to consider isopioids, where fentanyl is an extremely lipid-soluble opioid with an important Vd, whereas its activities are dosage dependent. After a bolus, fentanyl shows a fast onset and offset due to redistribution in the marginal tissues and an enhanced clearance in patients who are obese [12]. Alfentanil offset after several dosages or infusions, however, has a low inherent clearance. After bolus dosages, the alfentanil plasma concentration should be decreased due to enhanced cardiac output [13]. Unlike remifentanil, it differs in its pharmacokinetics because it experiences rapid and extensive tissue metabolism. Several studies conducted on athletes who were obese reported that the pharmacokinetics of remifentanil revealed that dosages based on the TBW could cause cardiovascular depression; hence, the LBW must be used to calculate the remifentanil dosage [14]. Morphine is a classic opioid with a slow offset and moderate onset of action. One of its metabolites is morphine-6-glucoside, a potent active compound. Individuals with obesity have a higher risk of respiratory depression associated with opioids due to concurrent disruptive sleep apnea and risks of accrual of both morphine and its active metabolite [2].

The metabolism of the neuromuscular blocking agent succinylcholine is mostly independent of organ function. However, decreased pseudocholinesterase due to hepatic dysfunction could slow metabolism. Hence, among individuals who are obese, if FW, LBW, and the plasma amount increase, dosing by TBW ensures that a suitable dose is provided as it accounts for enhanced pseudocholinesterase action [15].

Local delivery and binding of local anesthetics are influenced by dietary conditions, and enhanced α-acid glycoprotein can decrease the local anesthetic-free fraction and enhance the dosage requirements for nerve blocks among individuals with obesity, whereas central neuraxial block could be unpredictable due to obesity-related physical effects on the epidural space [16].

A volatile agent’s effect-site concentration depends on the pulmonary uptake, cardiac output, and inspired anesthetic agent fraction. Among individuals with obesity, functional residual capacity is decreased, cardiac output is enhanced, and the poorly vascularized borderline compartment is increased. Predictably, high pulmonary uptake is then offset by enhanced cardiac output. Furthermore, the effects of the enhanced marginal compartments were found to be alleviated through the volatile agent’s comparatively poor vascularity. Lipid-soluble agents for protracted procedures could accumulate and have an extended offset time; however, for minor procedures, the difference between athletes who were obese and non-obese was insignificant [17].

Pharmacodynamics of anesthetic drugs

The pharmacodynamics of anesthetic drugs in athletes can differ from those in the general population due to the unique physiology of athletes and the specific demands of their sport [18-20].

Inhalational anesthetics, such as desflurane, sevoflurane, and isoflurane, are commonly used in anesthesia. These drugs produce their effects by interacting with receptors in the central nervous system (CNS). The mode of action of inhalational anesthetics is not completely understood; however, they are thought to act by enhancing the activity of inhibitory neurotransmitters, such as gamma-aminobutyric acid, and by decreasing the activity of excitatory neurotransmitters, such as glutamate [21-23].

In athletes, the use of inhalational anesthetics may be complicated by several factors; one is the athlete’s cardiovascular system. Athletes often have larger hearts and greater cardiac output than those of nonathletes, which can affect the distribution and elimination of inhalational anesthetics. Additionally, athletes may be more susceptible to the cardiovascular depressant effects of inhalational anesthetics, which can cause hypotension and arrhythmias. Another factor that can affect the use of inhalational anesthetics in athletes is their body composition. Athletes often have lower body fat percentages than those of nonathletes, which can affect the distribution and elimination of inhalational anesthetics. The use of inhalational anesthetics in athletes with low body fat percentages may require adjustments to the dosing regimen to ensure adequate anesthesia [24-26].

Intravenous anesthetics, such as propofol, etomidate, and ketamine, are commonly used in anesthesia. These drugs work by interacting with CNS receptors. Intravenous anesthetics are administered by injection and produce rapid onset and offset of anesthesia, making them useful for inducing anesthesia [22,27,28]. Among athletes, the use of intravenous anesthetics may also be complicated by several factors. One factor is the athlete’s muscle mass. Athletes often have greater muscle mass than nonathletes, which can affect the distribution and elimination of intravenous anesthetics. Additionally, the use of intravenous anesthetics in athletes with greater muscle mass may require adjustments to the dosing regimen to ensure adequate anesthesia [29-31].

Another factor that can affect the use of intravenous anesthetics in athletes is their hydration status. Athletes may be more prone to dehydration due to the demands of their sport, and dehydration can affect the distribution and elimination of intravenous anesthetics. The use of intravenous anesthetics in dehydrated athletes may require adjustments to the dosing regimen to ensure adequate anesthesia [29-32].

Inhalational and intravenous anesthetics are commonly used in anesthesia, but their use in athletes may require adjustments to the dosing regimen to ensure adequate anesthesia. Clinicians must be aware of these factors when administering anesthesia to athletes to ensure safe and effective anesthesia [33-42].

Anesthetic implications of performance-enhancing drugs (PEDs)

PEDs are used by some athletes to build extra muscle mass.

Anabolic steroids (ASs) are made from cholesterol and are commercially available as oral and intravenous preparations [43]. These drugs are popular among weight lifters, bodybuilders, and sprinters and are the same in structure as the male sex hormone testosterone; hence, they boost male reproductive and secondary sex attributes. Consequently, patients can develop acne, virilization, and feminization. Further, typical sex function is changed among males, causing sterility, gynecomastia, and impotence. The ASs enhance muscle mass and strength by inspiring new muscle as well as cell growth, enabling athletes to become stronger. However, ASs also unfavorably affect the liver and cardiovascular system. Relevant cardiovascular alterations include secondary polycythemia, left ventricular (LV) hypertrophy (LVH), cardiac muscle fibrosis, hypertension, and cardiomyopathy. The risks of cerebrovascular events and myocardial infarction are also elevated [44].

The preoperative evaluation must start by recognizing probable AS abusers, such as those who have thick necks or extreme muscle mass. Athletes typically will not easily agree to provide information. Therefore, it is very important to inquire regarding steroid cycling and related drug use, either for recreational purposes or to mitigate the side effects due to steroid usage (Table 1) [43].

**Table 1:** Anabolic steroid cycling regimes Credit: Permission to reproduce this table has been obtained from reference [43].

Steroid dosing regimes			
Regime type	Duration	Description	Benefits
Cycling	4-12 weeks	Complete abstinence from steroid use between cycles	Minimize side-effects
Stacking	4-12 weeks	Use of more than one steroid per cycle (with complete abstinence between cycles)	Avoid tolerance
Pyramiding	4-12 weeks escalating dose	Cycling starts with a normal therapeutic dose and increases in increments 100 up to 1000 times	The dose is slowly reduced towards the end of a cycle to avoid withdrawal

During a steroid cycle, the use of artificial luteinizing hormone (LH) analogs (LHA), for example, tamoxifen, antagonizes estrogen receptors, increases testosterone levels, reduces water retention in the muscles, and prevents gynecomastia. In between cycles, LHA encourages testosterone levels to increase to their baseline faster. Preservation of baseline testosterone is important in maintaining muscle mass, as steroid cessation can inhibit testosterone production by 50% [43].

Special attention should be given to the neck thickness, airway, and tongue size. There is a need to inform patients regarding the risks related to anesthesia [43]. Investigations must include regular blood analyses, especially to identify electrolyte imbalances and irregular liver functioning as well as polycythemia resulting from the use of steroids and related drugs [43]. The overgrown deltoids and neck muscles could limit neck movement and cause trouble during intubation and bag-mask ventilation. An excessive muscle mass will cause an elevated oxygen consumption rate, and patients could desaturate more quickly than predicted. The weight of the patient’s muscle could decrease thoracic compliance and hence make the ventilation complicated [43]. These athletes may have enhanced requirements for oxygen and anesthetics. A few of these patients may be resistant to the nondepolarizing neuromuscular blockers caused by a rise in the nicotinic receptors. Physiological bradycardia can occur, and it could be helpful to keep a prophylactic anticholinergic, such as glycopyrrolate, readily available [43]. Extubation and emergence must be taken seriously, as patients using ASs are strong physically and may have a risk of psychotic episodes. It is recommended that they use a bite block, and in case of laryngospasm, there is a possible danger of negative pressure pulmonary edema. Therefore, gently and gradually awakening the patient is crucial [45].

The need for analgesics can vary among these athletes. Theory suggests that training can potentially raise the pain threshold; therefore, there are fewer analgesic requirements. Alternatively, high levels of circulating endorphins discharged during exercise boost tolerance to the anesthetics, increasing the need for analgesics. These individuals tend to develop polycythemia and related dangers, thus requiring suitable prophylaxis for venous thromboembolism (VTE) as part of their treatment [43].

A correctly sized arterial pressure cuff must be selected. Additional safeguards regarding pressure points, prophylaxis against deep vein thrombosis (DVT), and the consequences of manually handling these patients are also extremely important. Athletes with considerable muscle mass also could have an enormous potassium discharge after limb tourniquet deflation and could need treatment that includes fluids, calcium, and insulin dextrose [43].

Human chorionic gonadotrophin (hCG) and LH are frequently used together with ASs to either boost muscle building or counteract the adverse effects of elevated estrogen elevated. Once used with ASs, hCG increases testosterone and muscle growth among males. In both sexes, LH has an important role in maintaining normal testosterone levels, but it is used by weightlifters as an anti-aromatization treatment. Artificial LHAs, such as estrogen receptors and tamoxifen, can both antagonize and enhance testosterone levels. Generally, users tolerate them well, and there are some implications for anesthesia [43].

Human growth hormone (HGH) is produced naturally by the pituitary gland and is considered most significant for adolescent growth because it promotes protein synthesis and bone growth while reducing body fat by stimulating adipose tissue degradation. The hemoglobin glycation index is an important PED because it is not easily identified. Insulin-like growth factor 1 (IGF-1) also promotes protein and bone synthesis. Insulin has a significant role in the metabolism of protein, fat, and carbohydrates and could be used along with HGH or ASs to encourage muscle mass through the additional stimulation of protein synthesis. These artificially increased hormone levels are harmful to health [46].

Athletes may experience additional complications due to acromegaly. Blood glucose and thyroid function should be investigated before surgery, and blood glucose should be monitored intraoperatively [43].

Clenbuterol, terbutaline, and salbutamol are beta-2 (β2)-agonists being renowned therapeutic drugs primarily used for their bronchodilation effects. Some β2-agonists, such as fenoterol and clenbuterol, have added anabolic effects supposedly mediated through β2-receptors, but the precise mechanism has not been fully elucidated. A consistentlyhigh dose of oral salbutamol boosts endurance performance and muscle strength as well [47]. Because of these benefits, abuse is commonly observed; thus, all β2-agonists have been prohibited by WADA with the exception of inhaled salmeterol and salbutamol when used for therapeutic reasons [43].

The adverse effects of β2-agonists are well recognized and comprise tachyarrhythmias, lactic acidosis, hypokalemia, and prolonged QT. Generally, the toxicity is related to high-dose digestion and needs to be treated along with proper intravenous fluids, bicarbonate, potassium supplementation, and β-blockers preoperatively [48].

Amphetamines are sympathomimetic amines, known as amphetamines, which were first synthesized in 1887, but their popularity surged during World War II when used to combat fatigue and enhance alertness among military troops. Most athletes use amphetamines during competition, whereas wrestlers and gymnasts use them to repress appetite and decrease weight gain [43]. It is essential to distinguish between long-term and recent users; thus, a thorough examination of their history must be conducted separately for each [43]. Enhanced anesthetic requirements have been linked to acute amphetamine use, which has also been associated with increased intracranial pressure during surgery. However, constant users need considerably less anesthetic amounts supposedly because of CNS catecholamine reduction [49]. Postoperative development of refractory/unexpected hypotension and late development of serotonin syndrome are possible [43].

Endurance athletes, especially cyclists, frequently use caffeine, which can reduce their one-hour time trial result by up to 90 seconds [50]. Caffeine ingestion decreases the reaction time while increasing concentration. Caffeine boosts muscle spasms and enhances the exhaustion time by glycogen sparing. The drug also leads to diuresis which could help in obscuring other PED usage [43].

Cocaine is another common recreational drug used by athletes as a PED. Athletes experience a feeling of euphoria and heightened alertness after taking cocaine. Recurrent use causes tachyphylaxis, necessitating stronger fixes. It can also affect coordination and lead to anxiety, restlessness, and cardiac arrhythmias [43]. Although during surgery, patients may experience hypertension, arrhythmias, and tachycardia [43].

To increase oxygen delivery to exercising tissues, recombinant human erythropoietin (rHuEPO) is widely used by many endurance athletes, including marathon runners and cyclists. Erythropoietin increases erythropoiesis in the bone marrow while increasing the red blood cell density, thus enhancing the oxygen-carrying volume and enhancing it by 7% to 10%, and can be identified in the urine and blood of athletes; however, it is removed from the body in a short period making identification challenging [43]. A thorough history must recognize the probable complications of utilization. The investigations must comprise a complete blood count (CBC), electrocardiography/gram (ECG), and electrolytes. It is very important that these patients are fully hydrated preoperatively and given suitable VTE prophylaxis after surgery [43].

Blood doping refers to the act of introducing total blood into the body of an athlete to boost their capacity for carrying oxygen and produces the same effect as training at elevated altitudes. Therefore, it is widely used by long-distance event athletes, marathon runners, and cyclists [43]. Other athletes who need to maintain a steady hand while competing in sports often use relaxants [43]. β-blocker use is common among shooters, archers, and ski jumpers because of their anxiolytic and anti-tremor activities [43].

The recreational use of cannabinoids is unacceptable. These compounds are found in marijuana plants that include tetrahydrocannabinol (THC), which has psychoactive properties. THC is eliminated slowly and can persist in tissues for several weeks. Cannabinoids boost the hypnotic/sedative effects of several other CNS sedatives and may lead to cross-tolerance with opioids, benzodiazepines, phenothiazines, and barbiturates [51].

For masking the use of PEDs, diuretics are commonly used by judo, horse racing, rowing, wrestling, and boxing athletes because they stimulate PED excretion and can also be used to encourage rapid weight loss [43]. Epitestosterone is a type of testosterone that occurs naturally and does not provide any performance-enhancing effect. The use of epitestosterone injection by an athlete can decrease their epitestosterone/testosterone ratio, thereby concealing the usage of testosterone [43]. The clinical treatment of gout involves the use of secretion inhibitors that have structural similarities with organic acids and are eliminated through transport proteins inside the kidney. Transport protein is blocked, and drugs (PEDs) appearance in urine is prevented by them [43].

Food supplements and protein shakes are legally allowed and used by almost 76%-100% of athletes when compared with 50% of the general population. It is not recognized whether they boost performance. The unfavorable outcomes are also unknown [43].

Medical considerations for athletes undergoing anesthesia

Cardiac Considerations

Athletes belong to a distinctive group in which normal physical conditions can mimic disease [52]. Therefore, it is advisable to deliberate on the cardiovascular adjustments linked to athletic training, which will call attention to the medical conditions that need to be considered and closely assessed when administering anesthesia to athletes. An analysis of sudden cardiac death (SCD) among athletes revealed that hypertrophic cardiomyopathy (HCM) was the most prevalent cause, accounting for 26.4% of cases. Commotio cordis is the second most common cause at 19.9%, followed by coronary-artery anomalies, which account for 13.7% of cases. Cases of LVH of indeterminate causation account for 7.5% of SCDs. Less frequent causes include myocarditis (5.2%) and rupture of an aortic aneurysm associated with Marfan syndrome (3.1%) [53].

**Figure 2:**
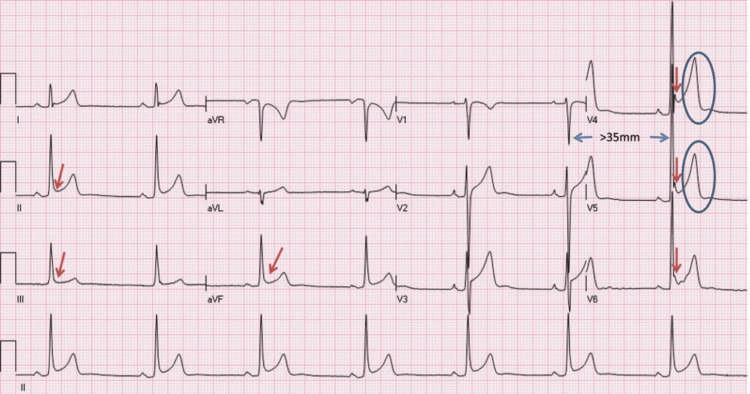
The ECG of a 29-year-old male asymptomatic soccer player demonstrating sinus bradycardia (resting heart rate of 44 bpm) This ECG also demonstrates early repolarization in I, II, aVE V2 to V6 (arrows), voltage criterion for LVH (S-Vl 1 RVS > 35 mm), and tall, peaked T-waves (circles). LVH: left ventricular hypertrophy Credit: Permission to reproduce this image has been obtained from reference [55].

Athletic heart conditions are referred to as physiologic alterations and cardiovascular adjustments related to intense athletic training, particularly bradycardia and ventricular dilation. The Swedish doctor Henschen first reported in 1899 that cardiac expansion in Nordic cross-country skiers was identified by percussion and auscultation. It became evident that athletes who experienced intense combinations of stamina training underwent cardiac remodeling, in contrast to nonathletes [54]. Regarding repeated systemic oxygen deficiencies among athletes, the LV cavity enlarges along with LVH to uphold cardiac output. Notably, the LV systolic function is generally normal. The manifestations of such structural adjustments can be identified by magnetic resonance imaging (MRI), transthoracic echocardiography, and ECG (Figure 2) [55].

**Figure 3:**
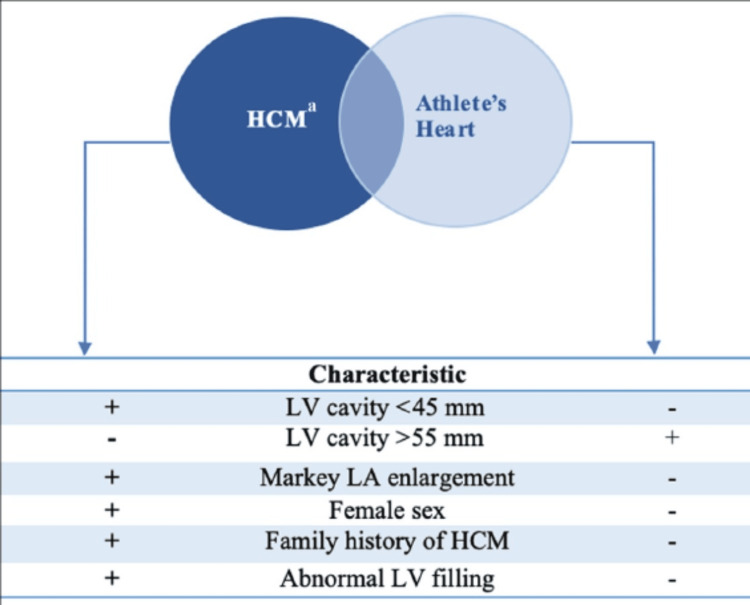
Differentiating HCM and physiologic cardiac changes in an athlete (athlete’s heart) HCM: hypertrophic cardiomyopathy; LV: left ventricular; LA: left atrium Credit: Permission to reproduce this image has been obtained from reference [56].

An athlete’s cardiovascular assessment must be carried out with thorough knowledge of structural and functional cardiovascular alterations related to severe athletic performance, particularly when trying to investigate certain cardiovascular conditions - for example, HCM, as several overlapping features are found between the natural pathologic and physiologic states (Figure 3) [56].

Hypertrophic Cardiomyopathy

HCM is a significant cause of SCD among young athletes and is found in approximately 1 out of 500 young adults (aged < 35 years) [57,58]. This disease must be at the forefront of investigative assessment to attain appropriate surveillance and treatment [52].

Regrettably, most deaths of famous athletes were due to HCM. It is important to mention that Reggie Lewis, a famous professional basketball player, passed away due to SCD at age 27. The autopsy results indicated that he had cardiomyopathy with significant myocardial scarring, indicating a possible diagnosis of HCM. In 2004, Miklos Feher, a Hungarian soccer player, experienced a lethal cardiac arrest, and autopsy results confirmed that the cause of death was HCM and ventricular tachycardia [59].

Acquiring medical clearance is vital for athletes with HCM before participating in sports activities. Notably, almost 25% of athletes who suddenly die had a significant cardiac disease identified or suspected before participation in sports [57]. In a study of sudden mortality among 158 athletes aged ≤40 years, 90% collapsed during or instantly after the training session, with 36% of athletes meeting HCM criteria on postmortem autopsy [53]. Physical examination is also a fundamental component, especially during the assessment of asymptomatic athletes with HCM [60].

The ECG evidence of HCM is characterized by a hypertrophied, but nondilated, LV without another condition that could cause such hypertrophy. The LV wall thickness in these cases can vary significantly, ranging from mild hypertrophy (13-15 mm) to massive hypertrophy (>30 mm) [52]. MRI could be of investigative value if the E reports are ineffective at recognizing segmental hypertrophy [61]. Among HCM patients, <75% will demonstrate an anomalous ECG although echocardiography fails to determine hypertrophy (Table 2) [52].

**Table 2:** Electrocardiographic evidence of left ventricular hypertrophy AVL: augmented vector left Credit: Permission to reproduce this table has been obtained from reference [52].

Electrocardiographic evidence of left ventricular hypertrophy
ST segment and T-wave changes
Large QRS complexes
High-voltage R waves in the anterolateral leads (V4, V5, V6, I, and AVL)

There is limited evidence indicating a significant correlation between the ECG patterns of LVH and HCM [60,62]. However, the physical examination and history of the patient could offer clues useful in further investigative assessments. The 2020 American Heart Association/College of Cardiology Guidelines regarding the diagnosis and treatment of patients with HCM suggest the following steps be performed [52]: (1) comprehensive cardiac history; (2) family history of three generations; and (3) comprehensive physical examination, including maneuvers such as squat-to-stand, Valsalva, walking, or passive leg raising.

**Figure 4:**
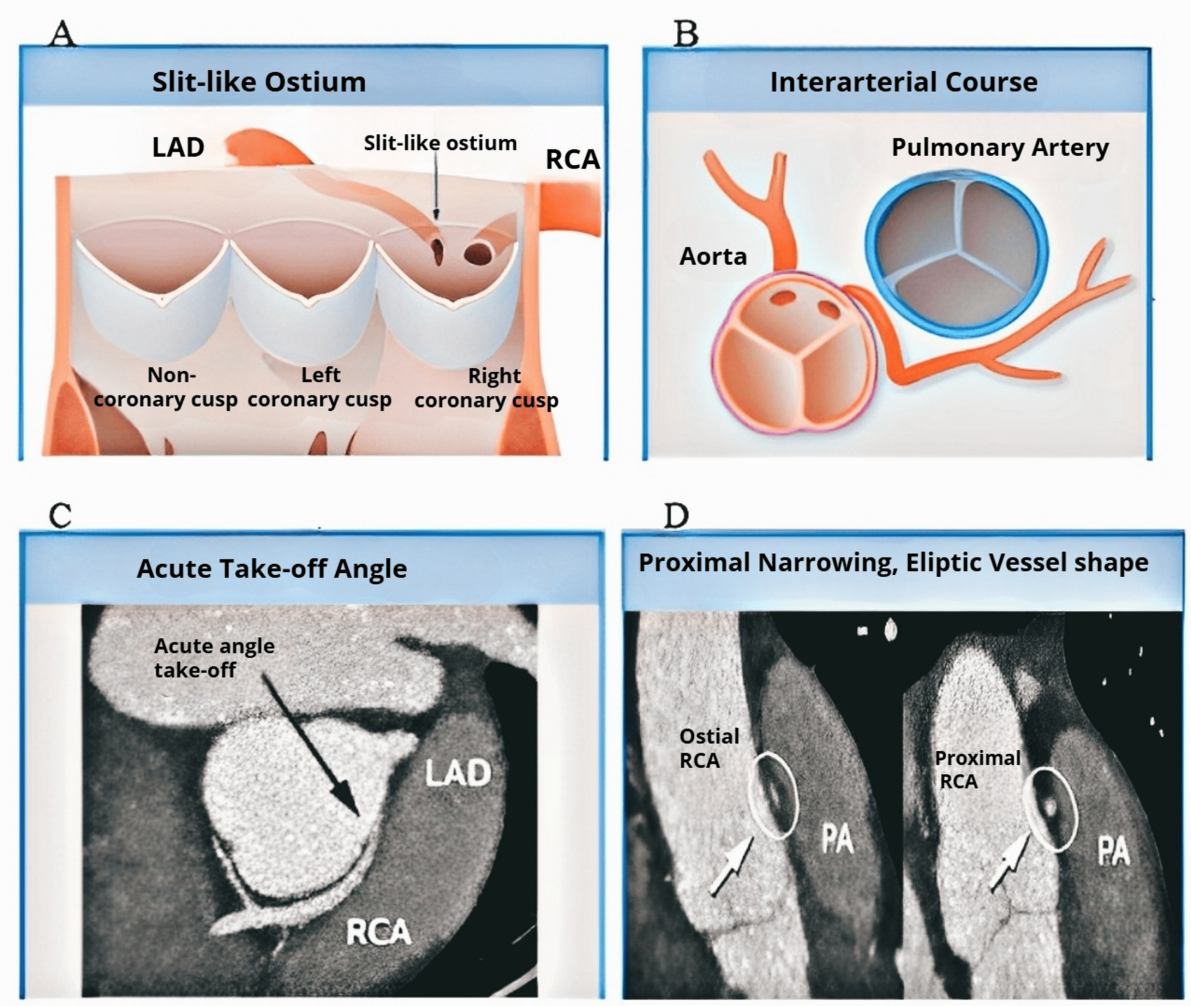
Anatomical features and variations in coronary artery anomalies. (A) Illustration showing a slit-like ostium, where the coronary artery’s orifice is elongated compared to the normal rounded ostium of the right coronary artery. (B) Depiction of an interarterial course of the coronary vessel, passing between the aorta and the pulmonary artery. (C) Acute take-off angle of the coronary artery, with the proximal segment emerging at an angle of <45° and a tangential course. (D) Proximal narrowing of the vessel, characterized by >50% narrowing of the proximal cross-sectional diameter and an elliptic shape (height/width ratio > 1.3), often associated with segmental hypoplasia LAD: left anterior descending; RCA: right coronary artery; PA: pulmonary artery Credit: Permission to reproduce this image has been obtained from reference [64].

Although among athletes, the congenital anomalous coronary artery is the second most common cause of SCD, responsible for 17% of mortality among young athletes in the USA [57,63]. This disease has two types: the most frequent type involves the left coronary artery coming from the right, or anterior, Valsalva sinus; however, in some patients, the right coronary artery comes from the left sinus [57]. A study conducted in 1974 found that 27.3% of the respondents among whom the left and right coronaries derived from the anterior sinus experienced SCD. The study results suggested that the coronary artery’s severe leftward passage alongside the aortic wall causes the left coronary system entrance to become slit-like (Figure 4) [64].

Once the coronary sinus is prone to aortic enlargement, for example, when enhanced cardiac output is observed during exercise, flaxlike closing of the orifice elongates, causing acute ischemia and fatal arrhythmia [64]. When assessing athletes, it is crucial to know their underlying heart condition because some individuals show no symptoms, and the majority still undergo SCD during or directly after physical activity [65]. In 2000, a review of two databases of young individuals showed that 55% of the athletes who passed away abruptly due to abnormal coronary arteries did not show noticeable cardiovascular symptoms during the examination. Athletes who experienced premonitory indications experienced chest pain and syncope. All cardiovascular examinations, such as ECG, maximum-exercise stress ECG, and 2D ECG for LV wall movement and cardiac dimensions, showed normal results [66].

Pulmonary Considerations

However, there seems to be a dissimilarity between exercise-induced asthma and exercise-induced bronchospasm (EIB); the difference is always unclear. Hence, these phrases are mostly utilized interchangeably [52].

This condition causes severe transient narrowing of the airway during exercise in 10%-50% of cases, mostly among those engaged in cold-weather sports [67,68]. The symptoms of EIB are chest tightness, coughing, wheezing, and shortness of breath that can only be activated through exercise without asthma’s typical features. This transitory narrowing of the airway is an intricate, multifaceted process that is mostly caused by airway drying due to an elevated exercise ventilation rate. According to one theory, the inhalation of dry air results in water loss from the airway, which alters the osmolarity of the airway lining. Consequently, mast cells are activated and produce provocative bronchoconstrictor mediators [67]. It is believed that this pathophysiology differs considerably from asthma because there is less allergic swelling and hence less reaction to the steroids in this state of the disease [52].

The athletes at higher risk include those who compete in high ventilation/endurance sports, such as long-distance running, swimming, and cross-country skiing. Environmental triggers, such as chemicals in the ice rinks and chlorine in the pools, also make athletes susceptible to developing EIB [68].

Several athletes who have EIB usually demonstrate normal baseline pulmonary function, whereas spirometry will not show this fundamental disease state [69]. Hence, a bronchoprovocation examination is suggested (Table 3) [52].

**Table 3:** Techniques for diagnosing exercise-induced bronchospasm Credit: Permission to reproduce this table has been obtained from reference [52].

Techniques for diagnosing exercise-induced bronchospasm
Eucapnic voluntary hyperventilation
Decrease in forced expiratory volume in 1 second (FEV1) of 10% for positive result
Hypertonic saline challenge
Decrease in FEV1 of 15% for positive result
Methacholine challenge
Inhaled mannitol challenge test

Both pharmacological and nonpharmacological approaches have been used for EIB treatment and prevention [70]. Typically, the EIB remains undetected and requires an intense evaluation to accurately identify the symptoms related to EIB to avoid needless respiratory distress among athletes [52].

Considering the elevated incidence of underlying asthma among EIB patients, the asthma must be optimally controlled before elective surgical treatment. However, the majority of anesthetics have bronchodilator effects, and asthma patients during intubation are more susceptible to bronchospasm because of airway manipulation. Hence, it might be useful to avoid endotracheal intubation and general anesthesia (GA) when feasible [71].

Obstructive Sleep Apnea (OSA)

This condition is persistent and caused by recurrent upper-airway breakdown during sleep, which in turn causes apnea and nocturnal asphyxia, which then causes fluctuations in blood pressure, fragmented sleep, and enhanced activity of the sympathetic nervous system [72]. It is known that OSA occurs in 25% of females and <50% of males among the general population [72]. However, there is a disturbing prevalence of OSA among athletes that frequently goes undetected. A study conducted among 257 retired football players reported that 52.3% had sleep-disordered breathing [73]. The finding of another study found that sleep-disordered breathing was present in 14% of athletes and 34% of football players [74].

A thorough history of daytime sleepiness, including apnea, snoring, headaches, poor concentration, irritability, and moodiness, is indicative of OSA. The OSA risk factors are age >40 years, BMI >35 kg/m^2^, male sex, neck circumference >16 inches, and hypertension [52].

A widely recognized and validated screening instrument, the “STOP-Bang questionnaire,” has become a frequently used method for identifying patients with OSA (Table 4) [75,76]. Several other screening examinations that have similar precision are the Berlin questionnaire, the Perioperative Sleep Apnea Prediction (P-SAP) score, and the American Society of Anesthesiologists checklist [77,78].

**Table 4:** STOP-Bang questionnaire to assess the risk of OSA Score 1 point for each positive response. Scoring interpretation: 0-2 indicates low risk; 3 or 4 indicates intermediate risk; >5 indicates high risk. OSA: obstructive sleep apnea Credit: Permission to reproduce this table has been obtained from reference [75].

STOP-Bang questionnaire to assess the risk of OSA	Yes	No
Snoring (Do you snore loudly?)	r	r
Tiredness (Do you often feel tired, fatigued, or sleepy during the daytime?)	r	r
Observed apnea (Has anyone observed that you stop breathing, or choke or gasp during your sleep?)	r	r
High blood pressure (Do you have or are you being treated for high blood pressure?)	r	r
BMI (Is your body mass index more than 35 kg per m^2^?)	r	r
Age (Are you older than 50 years?)	r	r
Neck Circumference (Is your neck circumference greater than 40 cm (15.75 inches)?)	r	r
Gender (Are you male?)	r	r

OSA has been associated with several postoperative complications, such as oxygen desaturation, difficulties with intubation during surgery, atrial fibrillation, cerebrovascular diseases (including an increased risk of stroke), and systemic and pulmonary hypertension. Thus, preoperative screening enables healthcare providers to use multimodal anesthetics as well as analgesics, methods, sparing narcotics, benzodiazepines, and GA whenever feasible to prevent obstructive symptoms exacerbation during the postoperative period [77].

Hematologic Considerations

Although preoperative laboratory analysis is not usually indicated, specific lab investigations are generally ordered for preoperative assessment, such as CBC, coagulation tests, and liver function tests. These investigations may show underlying anemia, coagulopathy, and thrombocytopenia that might warrant additional diagnostic workup [52].

It has been shown that 3.5% of males and 7.6% of females have anemia in the US population [79]. Most people who have mild anemia generally do not exhibit symptoms; however, athletes are more susceptible to the complications of anemia because their oxygen requirements are higher, particularly during physical exertion. The signs of anemia are fatigue, generalized weakness, syncope, and shortness of breath. Hence, it is important to know the reason for anemia to minimize needless and possibly incorrect inferences by physicians about an athlete’s performance. Iron deficiency can exist in the presence or absence of anemia. Unfortunately, ferritin is less sensitive for the diagnosis of iron deficiency without anemia (IDNA), and so IDNA is defined by low levels of ferritin (130/120 g/L in men/women). Athletes have an increased risk of developing iron deficiency through a variety of mechanisms (increased iron losses during training due to micro-ischemia, hemolysis, sweating, etc.) as well as women compared to men due to menstrual bleeding. The potential association between low energy availability (LEA) and low iron status in athletes could be due to suboptimal dietary intake of iron. The training-induced inflammatory response, characterized by both elevated IL-6 and hepcidin, creates a window wherein less iron is absorbed and recycled [80].

Venous Thromboembolism

Athletes can be prone to DVT under certain conditions, despite their generally good cardiovascular health, if any of the risk factors occur including dehydration, traumas, post-injury immobilization, the use of PEDs, and compression of blood vessels due to repetitive movements.

Thrombosis is a clot formation in a blood vessel, whereas clot migration is termed an embolism. Among postoperative patients, a typical concern is the consequent development of a pulmonary embolism (PE) and DVT due to endothelial impairment of blood vessels during an operation and consequent venous stability during the recovery period, indicated by two out of three Virchow triad components. Endothelial damage modifies blood-flow dynamics and causes turbulent flow in the vessel. The collagen is uncovered, which activates the extrinsic coagulation cascade, causing platelet aggregation. Stasis, which arises from postoperative pain or limb molding, enables the natural anticoagulant features of the blood to become affected as the blood slows, causing thrombus formation [81].

For DVT, physical examination is relatively unreliable, with typical results of asymmetric leg inflammation, warmth, and calf pain observed in <50% of patients. Patients with PE also might not have symptoms when 60% of the pulmonary circulation is blocked or present along with typical indications of right-heart failure, hypoxemia, and dyspnea in large occlusion settings. Hence, several scoring systems are available for estimating the pretest likelihood of both DVT and PE, with the Wells Criteria being the most prominent test (Tables 5-6) [82-85].

**Table 5:** Wells score for DVT Clinical pretest probability: score total 0 indicates low; score total 1-2 indicates intermediate; score total >3 indicates high DVT: deep vein thrombosis Credit: Permission to reproduce this table has been obtained from reference [84].

Clinical feature	Score
Active cancer (treatment or palliation within 6 months)	1
Bedridden recently >3 days or major surgery within 12 weeks	1
Calf swelling >3 cm compared with the other leg (measured 10 cm below tibial tuberosity)	1
Collateral (nonvaricose) superficial veins present	1
Entire leg swollen	1
Localized tenderness along the deep venous system	1
Pitting edema, confined to symptomatic leg	1
Paralysis, paresis, or recent plaster immobilization of the lower extremity	1
Alternative diagnosis of DVT as likely or more likely	-2

**Table 6:** Wells score for PE Clinical pretest probability: score total less than 2 indicates low; score total 2-6 indicates intermediate; score total >7 indicates high PE: pulmonary embolism; DVT: deep vein thrombosis Credit: Permission to reproduce this table has been obtained from reference [84].

Clinical feature	Score
Clinical signs and symptoms of DVT	3
Alternative diagnosis is less likely	3
Heart rate >100 bpm	1.5
Previous PE or DVT	1.5
Immobilization at least three days or surgery in the previous four week	1.5
Hemoptysis	1
Malignancy with treatment within six months or palliative	1

Endocrine Considerations

Athletes with DM can include those participating in youth sports to those competing at the Olympic level. Both present unique and complex challenges for themselves as well as for the healthcare professionals in managing their diabetes. Although 26.9 million individuals in America have type 1 diabetes mellitus (T1DM) or type 2 diabetes mellitus (T2DM), exercising is more difficult for those dependent upon insulin, which includes all persons with T1DM [86]. It is important that those who look after athletes with DM must investigate food habits, dietary supplement use, and PEDs, as these could have harmful effects on the management of glucose. For example, insulin nonadherence is a frequent practice among athletes in sports with weight categories, such as boxing or wrestling, in which athletes might wish to lose weight before their pre-competition weigh-in [87]. This certainly causes poor glycemic control and an enhanced risk of ketoacidosis [52]. All types of physical exercise also can have specific effects on DM, such as aerobic exercise that can cause hypoglycemia and anaerobic exercise bursts that can cause hyperglycemia [88]. These differences are significant for athletes undergoing anesthesia to have proper glucose control because hypoglycemia could be missed among anesthetized patients. Furthermore, high blood sugar causes delayed wound healing and could cause increased wound infections during the perioperative period [52].

Nutritional Considerations: Nulla per Os (Aka Nil per Os or Nothing by Mouth) Guidelines

The main purpose of fasting is to reduce the perioperative pulmonary aspiration risk that is described as gastric contents aspiration occurring after anesthesia induction, during surgery, or during the immediate postoperative period. The 2015 standards and practice parameters established by the American Society of Anesthesiology Committee only pertain to healthy patients who are scheduled for elective procedures and do not apply to patients who undergo non-anesthesia procedures or those who solely require local anesthetics because their upper-airway protective reflexes remain unaffected and are not susceptible to aspiration. The guidelines for minimum fasting duration are two hours for clear liquids, four hours for breast milk, six hours for nonhuman milk, six hours for light meals (toast, clear liquids), and eight hours for regular/heavy meals (fried/fatty food, meat) [89].

Anesthetic considerations for athletes

Although the majority of athletes, by definition, are fit and healthy, common medical problems arise during pre-anesthesia clinics. Diabetes, asthma, and other endocrine issues must be optimized and examined before administering anesthesia to all patients. Optimization infrequently requires increasing current drug regimes, requesting additional examinations, and then acting on their results to ensure safe anesthesia. A referral to other specialties for analysis of previously unidentified pathology is infrequently needed [1].

Nevertheless, athletes may have risks of unique problems, such as arrhythmias and cardiac issues, and these risks could be difficult to identify and evaluate [90]. However, if they are not identified and fully evaluated, there could be disastrous consequences. Close communication with cardiologists and the screening department is important for gaining complete knowledge regarding cardiac examinations and care. A normal distribution of the results is not necessarily applicable to athletes who are mostly believed to be above the 95th percentile of the population for most physiological limits. Therefore, an expert judgment must be made to determine if the supposed “abnormal” outcomes are actually normal for these athletes [1]. In addition, athletes live in countries around the world, so complete knowledge regarding regional disease must be obtained to help provide safe and optimum care [1]. It is imperative to inquire about the history of drugs and medication, specifically the use of any substance that is banned. Certainly, doctor-patient privacy remains sanctified, but there is a need to know about the use of some substances, such as amphetamines, frusemide, erythropoietin, and ASs, for safety reasons before performing anesthesia [1].

The long-term effects of ASs can be particularly worsened under anesthesia, leading to psychiatric issues, including psychosis, aggression, and hypomania can manifest after anesthesia [1]. Hypercoagulability and polycythemia can manifest in cerebrovascular accidents and myocardial infarctions. The cardiac consequences of long-term steroid use include ventricular hypertrophy, myocardial infarction, diastolic dysfunction, cardiac arrhythmias, and SCD [91,92]. There can also be massive swings in blood pressure while under anesthesia along with the subsequent risk of intracerebral hemorrhage and cerebrovascular accident. Hepatic damage results in a persistent ailment and possibly worsens into severe encephalopathy upon contact with specific anesthetic agents. Hyperglycemia, sodium imbalance, insulin resistance, and inhibitory effects on late healing and resistant function could also exist as serious irreversible side effects of AS abuse. Therefore, all legal or illegal drug use must be explained fully, and the unpleasant effects should also be fully examined and reversed before administering anesthesia. Furthermore, the use of “accessory drugs,” such as growth hormone, thyroxine, insulin, and beta-blockers, must be evaluated and accepted. These also can have acute and life-threatening outcomes while under anesthesia [1].

Psychological state

Most athletes are worried about any time needed away from competing, the probable unfavorable effects of GA and regional anesthesia (RA), and the long-term complications arising from this. Although anesthesia is now very safe, adverse effects and complications still occur. Clinical psychologists have important roles in the preadmission clinic, where the psychological aspects regarding care and any immediate concerns can also be discussed and optimized [1]. Occasionally, athletes need the care of trainers, physiotherapists, team members, and doctors, as well as reassurance, timely information updates, and continuing discussion.

Choice of anesthesia

Anesthesia is usually performed by a joint decision between the patient and the anesthetist. Athletes prefer GA to avoid any possible nerve issues (neuropraxia/neuropathy) that might be associated with RA. The incidence of complications associated with RA is very low (0.04% and <3% after neuraxial and peripheral nerve blockade, respectively). These complications are mostly temporary and persist for only a few days to a few weeks. Although any transient “patch of numbness” might not be concerning to nonathletes, it could be devastating to athletes; hence, proper counseling is essential in each case [1]. The significant risks associated with RAs include failed technique, neuropathy, neuropraxia, neurotoxicity, neuraxial hematoma, and infection [1].

Perioperative care

The most frequent concern among athletes is their cardiovascular status, as the LV is mostly hypertrophied, accompanied by associated ECG alterations and a large stroke volume as well as bradycardias. These can cause arrhythmias and infrequent ventricular standstill, which is greater than observed for “anxiety” by experienced observers. It is advisable to remain ready in advance with medications, such as atropine, adrenaline, glycopyrrolate, or ephedrine that are immediately available at any time [1]. There are various well-documented incidents of SCD among footballers during matches, and it is a terrifying situation for which all anesthetists must be prepared to treat while providing care to athletes under GA [1].

Postoperative management

It has also been suggested that athletes have a transformed pain tolerance that differs from the tolerance of nonathletes. Currently, there are two theories about this [1]: (1) The athletes have less ability to tolerate the pain caused by the effect of their training and an increase in the amount of circulating endorphins during their training, which leads to downregulation of the nociceptors, and hence, there is a high requirement for analgesics after surgery. (2) The athletes have a greater ability to tolerate the pain caused by their training and an increased amount of circulating endorphins during their training. The same downregulation occurs, but after injury, there could be a reduction in the regular discharge of physiological endorphins caused by a lack of regular physical activity. This effect could lead to rebound upregulation of the receptors; hence, athletes may need insignificant analgesia caused by opioid sensitivity. This inconsistency is usually determined within a healthcare facility, and there is an important adjustable analgesic need among these athletes after any surgical treatment. Some athletes have a greater need for analgesia, whereas other athletes hardly ever require any amount of analgesics. Several drugs used during the peri-anesthetic phase are available in the banned list of WADA; thus, their usage, if the athletes are examined, will need an official medical report or a filled-out therapeutic use exemption form [1]. A main concern during the immediate period after surgical treatment is the requirement for athletes to return to rehabilitation as quickly as possible. The use of DVT prophylaxis is important if there is any chance of current steroid use, protracted immobility, or an extended surgical period. A thrombosis/pulmonary embolus could be lethal and, at the very least, delay total training by weeks or months after the surgery [1].

## Conclusions

Anesthesia management in athletes requires special considerations to ensure safe and effective outcomes. The preoperative assessment focuses on identifying any underlying medical conditions, medication use, and substance abuse. Intraoperatively, the use of inhalational and intravenous anesthetics may require adjustments to the dosing regimen due to the unique physiology of athletes, including their muscle mass and hydration status. The postoperative management must include adequate pain control and monitoring for potential complications, such as delayed recovery or postoperative nausea and vomiting. Communication and collaboration with the athlete’s team and support staff also are critical for successful outcomes, especially in the context of intense public scrutiny. By considering these factors, anesthesiologists can provide excellent care for athletes and ensure their safe return to competition.
